# Comparison of Different Self-Sampling Devices for Molecular Detection of Human Papillomavirus (HPV) and Other Sexually Transmitted Infections (STIs): A Pilot Study

**DOI:** 10.3390/healthcare10030459

**Published:** 2022-02-28

**Authors:** Illari Sechi, Cocuzza Clementina Elvezia, Marianna Martinelli, Narcisa Muresu, Santina Castriciano, Giovanni Sotgiu, Andrea Piana

**Affiliations:** 1 Department of Medical, Surgical and Experimental Sciences, University of Sassari, Padre Manzella Street 4, 07029 Sassari, Italy; illasechi@uniss.it (I.S.); narcisamuresu@outlook.com (N.M.); piana@uniss.it (A.P.); 2 Department of Medicine and Surgery, University of Milano-Bicocca, Cadore Street, 48, 20900 Milano, Italy; marianna.martinelli@unimib.it; 3 Copan Italia S.p.A., 25125 Brescia, Italy; santina.castriciano@copangroup.com; 4 Clinical Epidemiology and Medical Statistics Unit, Department of Medical, Surgical and Experimental Sciences, University of Sassari, Padre Manzella Street 4, 07029 Sassari, Italy; gsotgiu@uniss.it

**Keywords:** HPV (human papillomavirus), high-risk human papillomavirus (hrHPV), sexually transmitted infections (STIs), vaginal self-sampling devices, self-collected samples, clinical accuracy, acceptability

## Abstract

Background: Cervical cancer is the fourth most common cancer in women, and it is well known that high-risk human papillomavirus (hrHPV) infections are the necessary carcinogenic factors for the development of cervical tumors. Moreover, the interaction between HPV and other sexually transmitted infections (STIs) may increase the risk of cancer progression. Self-sampling has been demonstrated to represent a valid and well-accepted alternative, favoring women’s participation in screening programs. This study aimed to investigate the use of FLOQSwabs^®^ (FS) as compared to two other vaginal self-collection devices for the detection of hrHPV and other sexually transmitted infections. Methods: Cervical and vaginal self-samples were collected, using two different combinations of vaginal self-sampling devices, from 40 women referred to colposcopy for a documented abnormal Pap smear. All samples were tested for hrHPV and seven STI pathogens using two commercial molecular assays. Results: Data on hrHPV detection from the first group of women showed an almost perfect agreement (kappa: 0.89) between cervical vs. FS vaginal self-samples, and a substantial agreement (kappa: 0.79) between cervical and HerSwab™ (HS) samples. In the second group of women, an almost perfect agreement (kappa: 0.90) was demonstrated in the detection of hrHPV between cervical samples vs. FS, and a moderate agreement (kappa: 0.60) for cervical vs. Evalyn^®^Brush (EB) self-collected samples. STI detections showed a very good agreement (kappa: 0.89 and kappa: 1.00) both among FS vs. HS and FS vs. EB, respectively. There was no statistically significant difference between the different devices used. The most frequently detected hrHPV genotypes in the studied population were HPV 16, 31, 35, 51, and 56; whilst the most frequently identified STI pathogens were *Ureaplasma parvum* and *Mycoplasma hominis.* Overall, investigated women did not report any discomfort in using the different vaginal self-collection devices. Conclusion: Evaluation of the three different vaginal self-collection devices confirmed their overall good acceptability by the studied population, as well as a similar agreement for hrHPV detection as compared to cervical samples. Our study indicated that the use of self-collected samples offers an alternative strategy to improve women’s participation in cervical cancer screening programs, but also underlined the importance of evaluating the concordance in hrHPV detection of collection devices in combination with the molecular hrHPV assay.

## 1. Introduction

Cervical cancer is the fourth most common cancer in women [1]. Although it represents a rare outcome of persistent infection with human papillomavirus (HPV) [2,3], the implementation and scale-up of screening and vaccination programs represent the most effective strategy to reduce its increasing burden worldwide. The Italian National Prevention Action Plan 2014–2018 recommended the replacement of the Pap test with an HPV test for cervical screening, as well as its adoption as a triage criterion for low-grade cytological anomalies [4]. However, poor adherence to screening programs is a major problem due to cultural, ethical, or religious factors. Vaginal self-sampling could overcome those barriers [5]; several countries are evaluating the inclusion of self-sampling in national screening programs [6]. Different vaginal self-sampling devices with different collection procedures, laboratory processes for analysis, and costs are commercially available.

Recent studies have indicated the potential role of sexually transmitted infections (STIs) in the development of cervical cancer in HPV-positive women [7]. Interactions between high-risk HPV (hrHPV) infections and other pathogens could accelerate cancer progression, enhancing HPV replication and persistence of infection. A high prevalence of STIs has also recently been reported in Italy in association with hrHPV and cervical dysplasia [8]. Wang and colleagues reported an association between *Ureaplasma urealyticum* (UU) subtypes and cervical cancer [7]; further studies reported that *Chlamydia trachomatis* (CT) increases the risk of cervical cancer [9,10]. Multiple STIs are therefore believed to represent a risk factor for cervical cancer [11]. Women have been shown to prefer vaginal self-collection for CT screening programs [12]. Moreover Graseck et al. showed that screening for *Chlamydia* and *Neisseria gonorrhea* through home collection is both feasible and acceptable to both men and women [13,14], including groups of marginalized populations [15,16,17]. Vaginal self-sampling could therefore represent a preferred and convenient way to screen women for both hrHPV and STIs from a single sample.

The aim of the present study was to investigate the agreement of hrHPV molecular detection by comparing clinician-collected cervical samples to self-collected vaginal samples obtained using different collection devices: FLOQSwabs^®^ (FS) (Copan Italia S.p.A., Brescia, Italy), Evalyn^®^Brush (EB) (Rovers Medical, Oss, The Netherlands), and HerSwab™ (HS) (Eve Medical, Toronto, ON, Canada). Detection of STIs from vaginal samples collected using the three different collection devices was also compared. Women’s acceptability of the use of the different vaginal collection devices was also evaluated.

## 2. Materials and Methods

### 2.1. Study Design and Sample Collection

Forty women with an abnormal Pap smear were enrolled at the Gynecology outpatient clinic of San Gerardo Hospital, Monza, Italy, between May 2018 and July 2018. The study protocol was approved by the Ethics Committee of the University of Milano-Bicocca, Italy (EC Approval 0037320/17; Prot. 305). Two vaginal self-collected samples followed by a clinician-collected cervical sample were collected from all enrolled subjects for the molecular detection of HPV and STI pathogens. The cytological assessment was performed according to the 2014 Bethesda System [18]. Cervical samples (CS) were collected using an L-shaped eso-endocervical FLOQSwab^®^ (Copan Italia Spa, Brescia, Italy) and transported to the laboratory following resuspension in 20 mL of ThinPrep^®^ PreservCyt^®^ Solution (HOLOGIC, Marlborough, MA, USA). Vaginal samples (FS, EB, and HS) were collected by the participating women prior to cervical sample collection at colposcopy. Specialized staff provided the patients with self-sampling devices, detailed self-sampling instructions, and a questionnaire about their experience on the ease of use, comfort, and satisfaction of the different collection devices. Vaginal self-collected samples were delivered dry to the laboratory and subsequently suspended in 5 mL of ThinPrep^®^ PreservCyt^®^ Solution (HOLOGIC, Marlborough, MA, USA) by swirling for 30 s before removing the swab prior to HPV molecular testing. For the Evalyn^®^Brush swab, before resuspension in ThinPrep^®^ PreservCyt^®^ Solution, the bristles of the brushes were cut with a sterile scalpel blade and then transferred to the elution tube.

**Figure 1:**
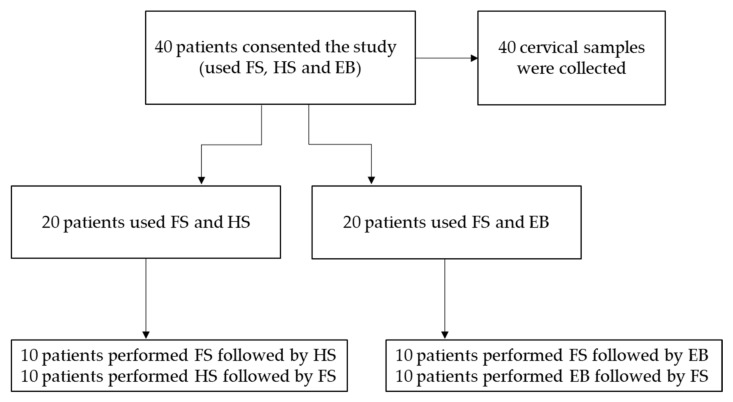
Summary of the work (flow chart).

Patients were divided into two groups (20 patients in each group). Women of the first group used FS and HS; in particular, 10 patients used the FS device followed by the HS, whilst 10 women used HS first followed by FS. The second group collected two self-samples using the FS and EB devices. Ten patients were asked to collect the first vaginal sample using the FS device followed by the EB; ten women used EB first followed by FS (Figure 1). The order of the device use was randomized. This protocol for the collection of two self-samples by each participating woman allowed us to minimize the number of variables between the matched self- and clinician-collected samples, thus minimizing potential bias.

### 2.2. DNA Extraction; HPV and STI Detection

DNA extraction was performed starting from 1 mL of resuspended cervical and vaginal samples using NucliSENS^®^ easyMag^®^ (bioMérieux, Marcy-l’Étoile, France), an automated system for total nucleic acid extraction from clinical samples. All samples were processed using the “specific B” protocol, characterized by a higher final elution temperature (70 °C) and the use of silica beads diluted 1:2, in accordance with the manufacturer’s instructions. The nucleic acid extracts were eluted in 100 µL of NucliSENS^®^ easyMag^®^ elution buffer (bioMérieux). HPV detection and typing were performed using the commercial assay Anyplex^TM^ I HPV28 (Seegene, Korea), which can detect 28 HPV types (i.e., 6, 11, 16, 18, 26, 31, 33, 35, 39, 40, 42–45, 51–54, 56, 58, 59, 61, 66, 68–70, 73, and 82) in 2 reaction tubes. Seven STI pathogens: *Chlamydia trachomatis* (*CT*), *Neisseria gonorrhoeae* (*NG*), *Trichomonas vaginalis* (*TV*), *Mycoplasma hominis* (*MH*), *Mycoplasma genitalium* (*MG*), *Ureaplasma urealyticum* (*UU*), and *Ureaplasma parvum* (*UP*) were also detected using AnyplexII™ II STI-7 (Seegene, Korea) from the same samples’ nucleic acid extracts. Real-time PCR assays were performed on the CFX96 real-time PCR instrument (Bio-Rad, Hercules, CA, USA). Data recording and interpretation were done using Seegene’s viewer software according to the manufacturer’s instructions.

### 2.3. Statistical Analysis

Cohen’s kappa value with SE of kappa and 95% confidence intervals was calculated to evaluate the degree of concordance in hrHPV detection on self-collected vaginal samples as compared to cervical samples. Kappa values were interpreted as follows: ≤0 showed no agreement; 0.01–0.20 none to slight; 0.21–0.40 fair; 0.41–0.60 moderate; 0.61–0.80 substantial; and 0.81–1.00 almost perfect. In addition, for each device, HPV and STI results were analyzed with a paired test (McNemar’s).

## 3. Results

### 3.1. Study Population

Forty patients with a previous abnormal cervical cytology (Pap test) result were referred to colposcopy. Their mean (standard deviation (SD)) age was 39.5 (11.16) years.

Reported referral cytology results were as follows: 40% (16/40) for LSIL and 40% (16/40) ASCUS, respectively; and 12.5% (5/40) HSIL, 5% (2/40) AGC-NOS, and 2.5% (1/40) ASCH. Colposcopy examination was abnormal in 10 patients (25%) (Table 1); they underwent a biopsy or conization, with 20% showing a cervical intraepithelial neoplasia grade 1 (CIN1), 40% a grade 3 (CIN3), 10% a cervical cancer, and 30% a negative result (Table 1).

### 3.2. HPV Detection and Typing

**Figure 2:**
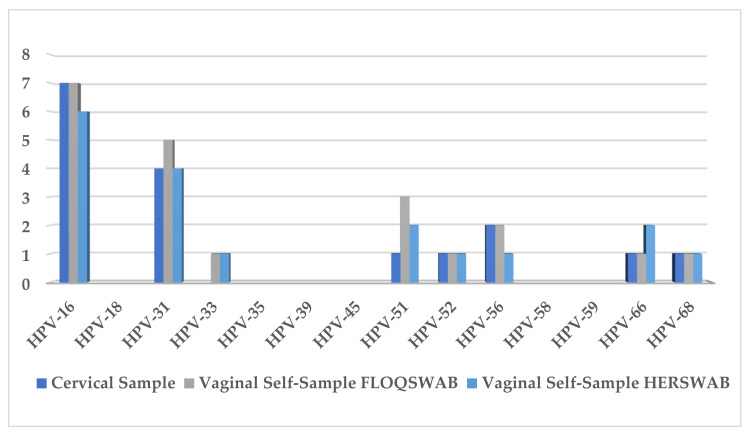
Prevalence of hrHPV genotypes from cervical samples and vaginal self-samples collected using FLOQSwabs^®^ (FS) and HerSwab™ (HS).

In the group of women that used FS vs. HS for vaginal sample collection, molecular data analysis showed that 60% (12/20) of cervical swabs were hrHPV-positive; 65% (13/20) showed positivity in vaginal samples collected using FS and 60% (12/20) with HS. Single hrHPV genotype infection was observed in 66.7% (8/12) of cervical samples, 53.8% (7/13) of FS and 50% (6/12) of HS; whereas multiple hrHPV infections were detected in 33.3% (4/12), 46.15% (6/13), and 50% (6/12) for cervical samples and using FS and HS, respectively. The most frequently detected hrHPV genotypes were HPV16, HPV31, and HPV51 (Figure 2).

An almost-perfect agreement (kappa: 0.89) was found between cervical samples and FS self-collection, whereas a substantial agreement (kappa: 0.79) was shown between cervical and HS. High *p*-values were found using McNemar’s paired test for HPV16 (*p* = 1.0) and HPV31/51/56 (*p* = 0.25).

**Figure 3:**
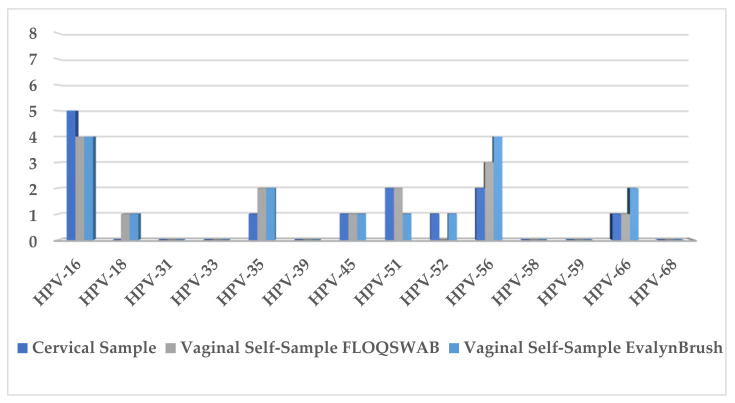
Prevalence of hrHPV genotypes for cervical samples and vaginal self-samples collected using FLOQSwabs^®^ (FS) and Evalyn^®^Brush (EB).

An hrHPV positivity of 55% (11/20), 50% (10/20), and 55% (11/20) was found in cervical samples, FS, and EB, respectively. Single and multiple infections were detected in 81.9% (9/11) and 18.2% (2/11) of cervical samples, 70% (7/10) and 30% (3/10) for FS, and 63.7% (7/11) and 36.4% (4/11) for EB. The most prevalent hrHPV genotypes were HPV16, HPV35, HPV51, and HPV56 (Figure 3). An overall agreement of 95% (kappa: 0.90) and 80% (kappa: 0.60) was found between hrHPV detection in cervical samples and vaginal samples collected using FS and for cervical samples and EB, respectively. For equivalence testing using McNemar’s paired test, *p*-values indicated equivalence for HPV16 (*p* = 1.0) and HPV31/51/56 (*p* = 1.0).

In women who were shown to have an abnormal histology biopsy, the hrHPV genotypes detected are shown in Table 2.

### 3.3. STI Prevalence

**Figure 4:**
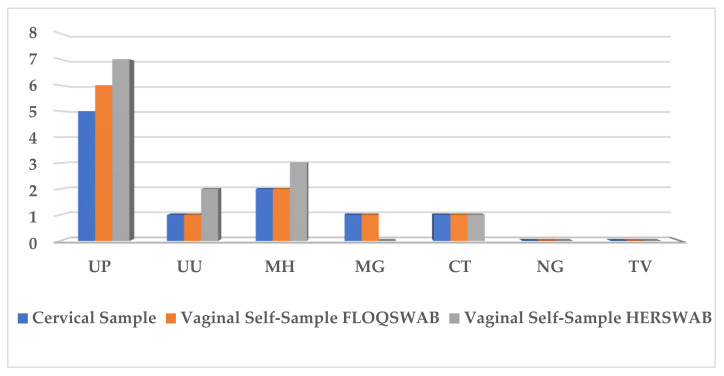
Prevalence of STI pathogens in cervical samples and vaginal self-samples collected using FLOQSwabs^®^ (FS) and HerSwab™ (HS). Seven STI pathogens were detected: Chlamydia trachomatis (CT), Neisseria gonorrhoeae (NG), Trichomonas vaginalis (TV), Mycoplasma hominis (MH), Mycoplasma genitalium (MG), Ureaplasma urealyticum (UU), and Ureaplasma parvum (UP).

A positivity for ST pathogens other than HPV was found in 35% (7/20), 40% (8/20), and 45% (9/20), corresponding to the first group of patients in cervical samples, FS, and HS, respectively. The most prevalent ST pathogens were *Ureaplasma parvum* and *Mycoplasma hominis* (Figure 4).

**Figure 5:**
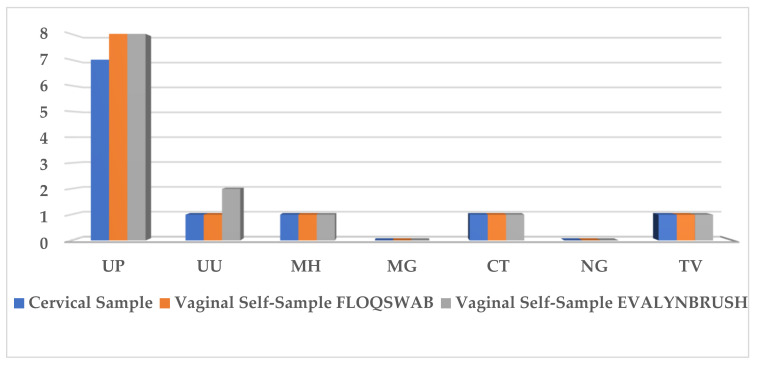
Prevalence of STIs pathogens in cervical samples and vaginal self-samples collected using FLOQSwabs^®^ (FS) and Evalyn^®^Brush (EB). Seven STI pathogens were detected: Chlamydia trachomatis (CT), Neisseria gonorrhoeae (NG), Trichomonas vaginalis (TV), Mycoplasma hominis (MH), Mycoplasma genitalium (MG), Ureaplasma urealyticum (UU), and Ureaplasma parvum (UP).

Data obtained from the second group of patients that used the vaginal self-collection devices (FS and EB) showed a positivity of 35% (7/20), 45% (9/20), and 45% (9/20) for the cervical samples, FS, and EB self-collection samples, respectively. The most frequently detected ST pathogen was *Ureaplasma parvum* (Figure 5).

An almost-perfect agreement was shown between FS-collected vaginal samples vs. HS (kappa: 0.89), and among FS vs. EB (kappa: 1.00), respectively. No discordant pairs were found with McNemar’s equivalence test (*p* = 1.0).

### 3.4. Patient Satisfaction

The analysis of satisfaction questionnaires relating to the use of the FS, HS, and EB vaginal self-collection devices for the 40 recruited women demonstrated a good overall degree of satisfaction. Almost all participating women considered the procedures of self-sample collection easy to perform. No patient reported experiencing pain while performing the self-test. The reinsertion of the swab into its container at the end of the collection was also deemed easy, and no reports of difficulties were recorded. The instructions provided for performing the self-collection were found to be clear and understandable by all patients, and the whole procedure was deemed easy with unanimous opinion (Table 3).

Almost all the patients said they would prefer to use vaginal self-sampling compared to having clinician-performed cervical sampling. Finally, in reply to the question: “Which self-collecting device do you prefer?”, the group of women using the FS and HB devices for vaginal self-collection reported a 40% (8/20) preference for the FS device and a 35% (7/20) preference for HS, while 25% (5/20) were indifferent. The second group of women expressed a 50% (10/20) and 30% (6/20) preference for the FS and EB self-collection devices, respectively, and 20% (4/20) were indifferent. An overall preference for the use of FS swabs was expressed by the women participating in the study.

## 4. Discussion

Participation of Italian women aged 25–64 years in cervical cancer screening (Pap test or HPV DNA test) was shown to be approximately 80% [19], with higher rates in married individuals and in those with higher incomes. However, the number of women who do not participate in cervical cancer screening remains high: some women feel that they are healthy and do not need to adhere to any screening program, whereas for some women, the need for a physician-collected cervical sample can represent a sociocultural and/or religious barrier. It is therefore important to implement new strategies to improve adherence to cervical cancer screening [5].

Self-collection of vaginal samples, based on noninvasive and easy-to-perform procedures, for the detection of HPV DNA detection could represent a more attractive alternative to increase the participation of hard-to-reach women in screening programs [20]. Published meta-analyses confirmed the accuracy of HPV DNA testing on self-collected versus clinician-collected samples, using PCR-based assays [21,22,23]. This screening strategy can be particularly useful in cervical cancer prevention in disadvantaged populations and/or in low-resource settings [24,25,26].

HPV DNA testing of self-collected samples therefore represents a feasible alternative, improving participation in cervical cancer screening; avoiding women’s embarrassment, discomfort, and anxiety; and allowing implementation of population screening programs in low- and middle-income countries where screening has not previously been a priority and/or where the medical infrastructure is less developed.

However, it is essential to demonstrate the diagnostic accuracy of the HPV test on self-collected samples by validating the collection device in combination with the specific PCR-based HPV assay. Furthermore, HPV assays including a human internal control can allow clinicians to evaluate sample adequacy, which may be of particular importance in testing self-collected samples.

The recently published VALHUDES protocol [27] aims to clinically validate different PCR-based HPV tests in combination with specific collection devices for self-vaginal and urine samples, compared to HPV testing performed on cervical samples taken by specialized personnel, in a colposcopy setting [28].

The aims of the present pilot study were to perform a preliminary evaluation of the analytical concordance of HPV and STI molecular testing in combination with some of the most frequently used vaginal self-collection devices (FS, HS, and EB), as well as to assess women’s acceptability of the use of the self-collection devices included in the study.

Our findings confirmed previous findings: self-collection of vaginal samples can provide good analytical concordance in molecular HPV detection as compared to that obtained from clinician-collected cervical samples. STI detection also showed a good concordance between vaginal samples collected with the three different devices.

The present pilot study reported the concordance in hrHPV and STI detection in self-collected vaginal samples using different devices.

The agreement of self-performed vaginal samples and clinician-performed cervical samples has been the topic of many studies [28,29,30].

Our results were consistent with the literature: performance of self-collection is determined by its constituting parts, including the collection device, hrHPV assay, and specimen preparation [29,31]. In a study by Ertik et al., EB showed a slightly higher concordance and higher proportion of positive agreement with physician-collected specimens (kappa: 0.48) than FS for all hrHPV assays (kappa: 0.29) [29]; while from the data obtained from our preliminary analysis showed a higher agreement with clinician-collected samples as demonstrated by FS (kappa: 0.90 vs. 0.60, FS vs. EB).

Regarding the HS device, the concordance result obtained in our study (kappa: 0.89 vs. 0.79, FS vs. HS) was higher than what was reported by Bokan and colleagues (kappa: 0.45 vs. 0.53, HS vs. Qvintip) [30].

Self-collection for the detection of STIs has been previously reported as a reliable and acceptable alternative to screening, and can reach marginalized populations [15,16,17].

Unfortunately, there are only few previous studies in the literature comparing different self-collection devices for the detection of STIs [32,33], so future studies are needed to evaluate this comparison. Even if an almost-perfect (kappa: 0.89; kappa: 1.00) agreement among FS vs. HS and FS vs. EB self-collected samples was shown for the STI detection, the pathogen detected in this study was too limited to come to any conclusions.

These results, however, need to be confirmed in a larger number of patients, as in general, one of the main drawbacks of this pilot study was the small sample size of the evaluated women. A recently published study protocol [27], for the validation of self-collection devices in combination with HPV tests reported a required sample size of 500 women referred to colposcopy for cytological abnormalities. Our pilot study was also performed in a similar colposcopy setting, which allowed for high HPV and STI positivity rates in the enrolled population. For future research, it would be advisable to plan studies based on the low estimated discordance we found in the case of planning of studies focused on the diagnostic agreement. For example, if we estimate a discordance rate of 5% between a cervical sample and the FLOQSwabs^®^ self-collection device following our findings, based on an alpha and beta error of 0.05 and 0.20, respectively, the sample size would be equal to 460, implying the need for a multicenter design.

Furthermore, the study design involved the collection of two vaginal samples by each participating woman. However, the order of vaginal sample collection, using the two different devices in each patients’ group, should not have affected the result of the analyses, as the molecular quantification of human cells, as a measure of the sample’s adequacy, showed similar values for all study samples, irrespective of the order of collection (data not shown). Moreover, equivalence testing using McNemar’s paired test showed high *p*-values, further confirming equivalence. McLarty and colleagues reported high *p*-values and 100% concordance between swab self-collected specimens and clinical-collected specimens [34].

Regarding laboratory procedures required for the testing of self-collected vaginal samples, processing FS- and HS-collected samples was easier to perform, whereas the EB swab is labor-intensive, requiring the bristles of the brush to be cut off with a sterile scalpel blade for resuspension of the vaginal sample in ThinPrep^®^ PreservCyt^®^ Solution. 

Women’s acceptability of the use of different commercially available collection devices can differ, as also reported by Ertik et al. and Mahomed et al. [29,32]. Moreover, Bishop and colleagues [35] indicated that the type of device may play a key role in women’s acceptability of self-sampling, as women tend to prefer simpler devices.

A good degree of satisfaction, in line with previous studies, was also more recently reported for self-sampling procedures [36,37]. Virtanen et al. [38] reported favorable experiences of “ease of sampling taking”, “no pain”, “less embarrassment”, and “less fear or anxiety” with self-sampling in >70% of subjects. The instructions provided for the execution of the self-collection were deemed understandable. As a whole, almost all screened women preferred self-sampling systems. FS self-sampling would be the preferred option when considering patient attitudes, sample processing activities, and cost.

## 5. Conclusions

Cervical cancer cases are still being diagnosed in “hard to reach women” who do not participate in cervical cancer screening. Improving the participation of women who do not adhere to screening programs should be a public health priority, and the results of our pilot study confirmed that the introduction of innovative self-collection methods offers a valid solution. FLOQSwabs^®^ proved to have a good acceptability by the studied population, as there was a good concordance in the detection of hrHPV and STIs as compared to cervical samples. Future larger studies are needed, however, to further evaluate the use of self-collection in cervical cancer screening in terms of both analytical and clinical accuracy.

## Figures and Tables

**Table 1:** Clinical results of the cohort of enrolled women.

**Cytology**	**HSIL**	***n* (%)**	**Total *n***
		5 (12.5)	40
	**ASCH**	1 (2.5)	
	**LSIL**	16 (40)	
	**AGC-NOS**	2 (5)	
	**ASCUS**	16 (40)	
**Colposcopy examination**	**Cervical abnormality**	10 (25)	40
	**Negative**	30 (75)	
**Histology**	**CIN 1**	2 (20)	10
	**CIN 3**	4 (40)	
	**Cervical Cancer**	1 (10)	
	**Negative**	3 (30)	

HSIL, high-grade squamous intraepithelial lesion; ASCH, atypical squamous cells—cannot exclude HSIL; LSIL, low-grade squamous intraepithelial lesion; AGC-NOS, atypical glandular cells—not otherwise specified); ASCUS, atypical squamous cells of undetermined significance; CIN 1, cervical intraepithelial neoplasia grade 1; CIN 3, cervical intraepithelial neoplasia grade 3.

**Table 2:** Number of hrHPV-positive cases in women biopsied, categorized according to cytology and histology.

Cytology	Histology	hrHPV Types Detected			
		Cervical Sample Collection	FS Vaginal Self-Samples	HB Vaginal Self-Samples	EB Vaginal Self-Samples
LSIL	CIN 1	66	51, 66	51, 66	-
AGCUS	Cervical Cancer	16	16	16	-
LSIL	CIN 1	16	16	16	-
HSIL	CIN 3	16	16	16	-
HSIL	CIN 3	16	16	16	-
ASCUS	CIN 3	52	31, 52	31, 52	-
HSIL	Negative	16	16	-	16
HSIL	CIN 3	16	16	-	16
ASCH	Negative	16	16, 18, 56	-	16, 18, 56
ASCUS	Negative	35	35	-	35

**Table 3:** Satisfaction questionnaire for FS, HS, and EB self-collection devices.

	FS (*n*: 40)		HS (*n*: 20)		EB (*n*: 20)	
	YES	NO	YES	NO	YES	NO
Did the swab cause discomfort during sample collection?	2 (5%)	38 (95%)	0 (0%)	20 (100%)	0 (0%)	20 (100%)
Did the swab cause bleeding during sample collection?	2 (5%)	38 (95%)	2 (10%)	18 (90%)	0 (0%)	20 (100%)
Were self-collection instructions clear?	38 (95%)	2 (5%)	18 (90%)	2 (10%)	19 (95%)	1 (5%)
Was self-collection acceptable and easy to perform?	40 (100%)	0 (0%)	19 (95%)	1 (5%)	20 (100%)	0 (0%)
